# Development and Validation of a Risk Predictive Model for Small Intracranial Aneurysms in Adults Over a Five-Year Period

**DOI:** 10.7759/cureus.67652

**Published:** 2024-08-24

**Authors:** Yiya Xu, Chao Chen, Yinzhou Wang

**Affiliations:** 1 Department of Neurology, Fujian Provincial Hospital, Shengli Clinical Medical College of Fujian Medical University, Fuzhou, CHN; 2 Fujian Key Laboratory of Medical Analysis, Fujian Academy of Medical Science, Fuzhou, CHN

**Keywords:** small, nomogram, predictive model, rupture, intracranial aneurysms

## Abstract

Objective

The optimal management of a small intracranial aneurysm (sIA) remains a challenge due to the lack of a size-specific risk predictive model for aneurysm rupture. We aimed to develop and validate a nomogram-based risk predictive model for sIA.

Methods

A total of 382 patients harboring 215 ruptured and 167 unruptured small intracranial aneurysms (uSIAs) (≤ 7 mm) were recruited and divided into training and validation cohorts. Risk factors for the construction of a nomogram were selected from clinical and aneurysmal features by least absolute shrinkage and selection operator (LASSO) and multivariate logistic regression. The nomogram for risk of rupture was evaluated in both the training and validation cohorts for discrimination, calibration, and clinical usefulness.

Results

Hyperlipidemia (odds ratio (OR)=2.74, 95% confidence interval (CI)=1.322~5.956, P=0.008), the presence of a daughter dome (OR=3.068, 95%CI=1.311~7.598, P=0.012), larger size-to-neck ratio (SN) (OR=1.807, 95%CI=1.131~3.063, P=0.021) and size ratio (SR) (OR=2.221, 95%CI=1.262~4.025, P=0.007) were selected as independent risk factors for sIA rupture and used for construction of nomogram. Internal validation by bootstrap sampling showed the Concordance index (C index) of 0.756 for the nomogram. The calibration by the Hosmer-Lemeshow test showed a P value of 0.847, indicating the model was well-fitted. Additionally, decision curve analysis (DCA) demonstrated that the predictive model has good clinical usefulness, providing net benefits across a range of threshold probabilities, thus supporting its application in clinical decision-making.

Conclusion

The risk prediction model can reliably predict the risk of sIA rupture, which may provide an important reference for optimizing the therapeutic strategy.

## Introduction

Rupture of intracranial aneurysm (IA) is the most important cause of subarachnoid hemorrhage (SAH), which is of high mortality and morbidity rates. Small intracranial aneurysm (sIA), which is usually defined as IA with a size of less than 7 mm in diameter, accounts for more than 60% of all IAs in the adult population and remains the source of challenge for making treatment strategies [1].

Early prospective studies on the natural history of unruptured IA have shown that the risk of rupture for small unruptured intracranial aneurysm (sUIA) (<7 mm）is low, thus conservative treatment instead of surgical clipping or endovascular coiling is suggested for patients with sUIA [2-4]. However, the practical incidence of SAH attributed to rupture of sIA is quite common. A 5-year retrospective study reported that 73% of the 131 ruptured IAs during the study period were associated with IAs that were smaller than 7 mm [5]. The other report involving 1256 aneurysmal subarachnoid hemorrhage (aSAHs) also showed that 47.1% of the cases were contributed by the rupture of IAs 2-5 mm in size [6]. Consensus has been not yet achieved worldwide regarding whether a surgical procedure or only conservative treatment should be performed for patients with sUIA. Due to the devastating outcome of SAH resulting from the rupture of IA, it is critical to identify and perform prophylactic clipping or coiling to those sUIA at high risk of rupture. It is observed in many studies that, apart from IA size, the risk of IA rupture was significantly influenced by many other factors including demographic features and IA morphology [7-9]. To better assess these risks and guide treatment decisions, there has been a growing interest in developing predictive models for sIA rupture. Recent efforts have been made to develop predictive models for sIA rupture based on the identified risk factors, which have shown prospective potential for guiding decision-making in clinical practice. Nevertheless, in these reports, different sizes were used for defining sIA, and the risk factors identified to establish the predictive model varied among the investigations, which indicates the need for further research [9-11].

Here, we aim to develop and validate a nomogram-based predictive model for sIA rupture by retrospective analyzing data of hospitalized sIA patients in a tertiary hospital. In this study, we define sIA as a diameter less than 7 mm since this is consistent with early studies [12]. We select risk factors from both the clinical features and characteristics of aneurysm morphology, comparing with those reported recently, which is important for consolidating the reliability of both the current and previously reported models.

## Materials and methods

Ethical considerations

This is an observational, case-controlled, retrospective study, which was approved by the Ethical Committee of Fujian Provincial Hospital (K2022-01-017). The collected data were guaranteed anonymity and confidentiality during the study period. Informed consent was waived by the institutional review board because of the retrospective design of the study.

Study design and patient enrollment

**Figure 1:**
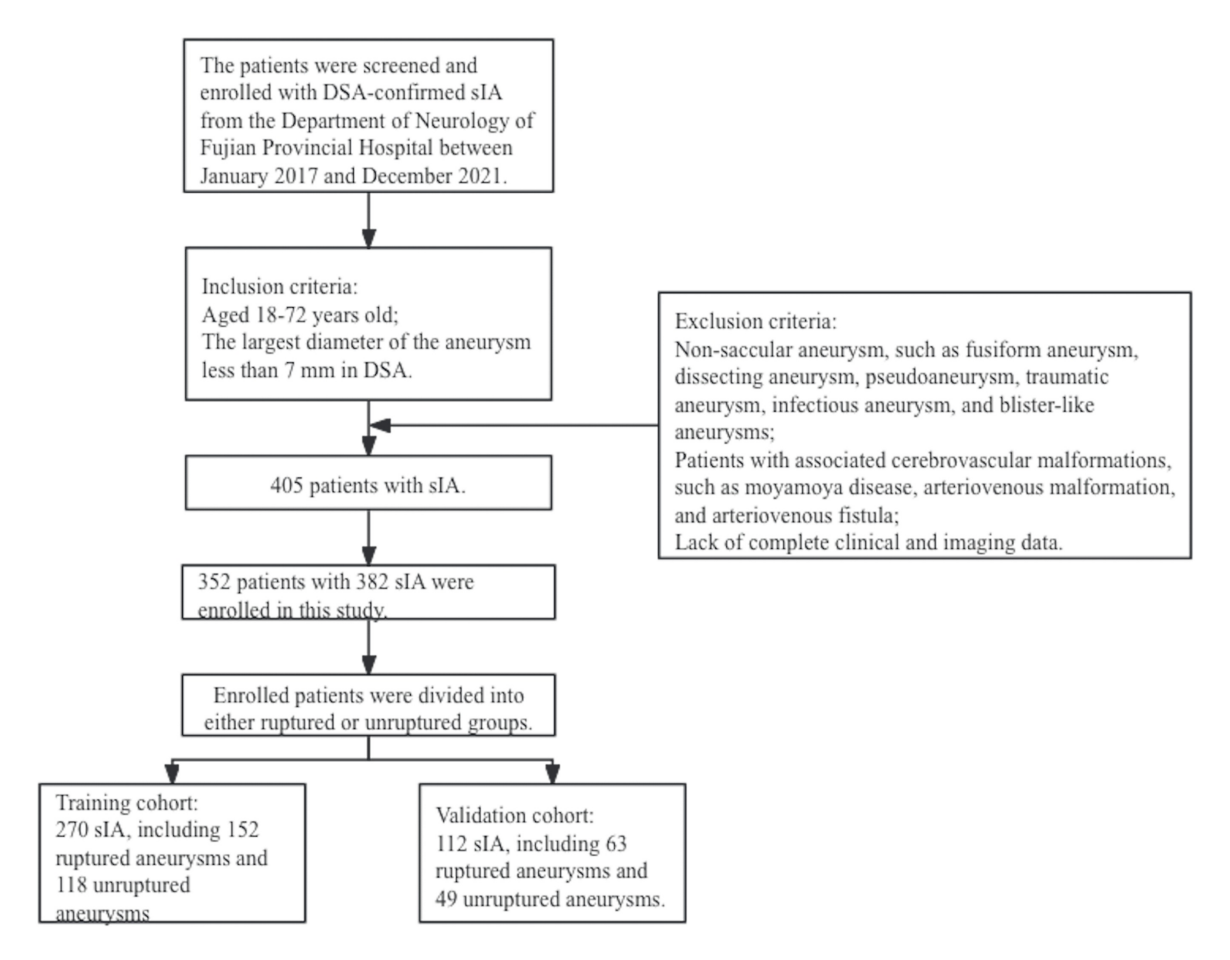
Flowchart of patient enrollment We enrolled patients with IA between January 2017 and December 2021. A total of 352 patients with 382 sIAs were ultimately included in this study. The training cohort (including 270 sIAs with 152 ruptured aneurysms and 118 unruptured aneurysms) was used to establish the predictive models. The validation cohort (including 112 sIAs with 63 ruptured aneurysms and 49 unruptured aneurysms) was used to verify the accuracy of different predictive models. The ratio of unruptured sIA in the primary cohort to the validation cohort was approximately 3:1. IA: intracranial aneurysm; sIA: small intracranial aneurysm

We screened and enrolled patients with digital subtraction angiography (DSA)-confirmed sIA who were admitted to the Department of Neurology of Fujian Provincial Hospital between January 2017 and December 2021 and completed the data collection in January 2022. The data were obtained from hospital electronic medical records. All patients included in the study were between 18 and 72 years of age. The sIA was defined as an intracranial aneurysm with the largest diameter of less than 7 mm in DSA. The rupture of sIA was diagnosed based on plain brain CT or lumbar puncture. sUIAs were found in patients undergoing cerebral angiography for various presentations or routine body checks who had no history of a cerebral hemorrhage. Patients with non-saccular aneurysms (ANs), such as fusiform aneurysms, dissecting aneurysms, pseudoaneurysms, traumatic aneurysms, infectious aneurysms, and blister-like aneurysms, were excluded. Patients comorbid with Moyamoya disease, arteriovenous malformation (AVM), or cerebral arteriovenous fistula were also excluded. Enrolled patients were divided into either the ruptured or the unruptured group. Finally, the study enrolled 352 patients with 382 sIAs from a total of 613 intracranial aneurysms. We randomly assigned the 382 sIAs to the training cohort and validation cohort at a ratio of 7:3. The clinical characteristics and features of aneurysm morphology were compared between the ruptured and unruptured groups (Figure 1).

Clinical features of patients

Clinical features, including age, gender, blood pressure at admission, and a history of hypertension, diabetes mellitus, coronary heart disease, hyperlipidemia, smoking, and drinking, were collected and compared between the ruptured and unruptured sIA groups.

Measurement of AN morphology

All patients underwent angiography using the ARTIS Icono Biplane (Siemens Healthineers, Erlangen, Germany) and biplane neurovascular imaging systems (Philips, Amsterdam, Netherlands), both equipped with data workstations and processing software to detect the presence of small intracranial aneurysms (sIA).

The morphological characteristics of each sIA were measured based on a 3D-reconstructed image of the AN by two senior neuro-interventionists who were blinded to the information on the group assignment of the patients. We collected data on aneurysm location (categorized into seven types: anterior cerebral artery, anterior communicating artery, internal carotid artery, middle cerebral artery, posterior communicating artery, vertebral artery, and basilar artery), as well as morphological characteristics and parameters. These parameters included aneurysm length (the distance from the center of the aneurysm neck to the dome), aneurysm width (the maximum width of the aneurysm perpendicular to the plane of the neck), neck width (the longest diameter at the junction between the aneurysm and the parent artery), aneurysm height (the maximum vertical distance within the aneurysm perpendicular to the plane of the neck), and parent artery diameter (the average diameter of the parent artery measured at the adjacent segment to the neck and at 1.5 times the diameter (D) proximal to the neck, referred to as D1). Aneurysm shape was classified as regular (smooth and spherical or ellipsoidal without lobulations) or irregular (with lobulations, sacs, fusiform or sausage-shaped aneurysms, or a rough surface; any of these features were considered irregular). Additionally, we recorded the presence of sacs or lobulations (Appendix A) [7,8,13]. We calculated several ratios and indices, including the size-to-width ratio (SW, defined as the ratio of aneurysm length to aneurysm width), the size-to-neck ratio (SN, defined as the ratio of aneurysm length to neck width), the size ratio (SR, defined as the ratio of aneurysm length to parent artery diameter), the aspect ratio (AR, defined as the ratio of aneurysm height to neck width), aneurysm volume (V), and aneurysm neck area (S). We then calculated the ratio of aneurysm volume (V) to neck area (S); all definitions and formulas are provided in Appendix B. Measurements were taken using electronic calipers with an accuracy of two decimal places (0.01 mm). Each parameter was measured three times, and the final value was determined by averaging the results obtained by two independent neurologists.

Statistics

All statistical analysis was performed using SPSS version 26.0 (IBM Corp., Armonk, NY, USA) or R (version 3.6). The S-W test was conducted to evaluate the distribution of continuous data. Continuous data of normal distribution and non-normal distribution were compared between the two groups by the unpaired Student t-test and the Mann-Whitney U test, respectively. The categorical data were compared by the chi-square test. All tests were two-sided with P≤0.05 indicating statistical significance for the difference.

To select the predictive factors for sIA rupture, we first screened the 26 clinical and morphological characteristics using LASSO (Least Absolute Shrinkage and Selection Operator) regression in the training cohort. The selected variables were further analyzed by multivariate logistic regression, in which variables with p<0.05 were selected as independent predictive factors for nomogram development.

The predictive performance of the model was evaluated by discrimination and calibration in the training cohort. The discriminatory ability of the nomogram was assessed based on receiver operating characteristic (ROC)-area under the curve (AUC) analysis. The calibration was determined by drawing a calibration plot curve, which visualized the consistency between the predicted risk and actual incidence of sIA rupture. The fitness of the model was further assessed by the Hosmer-Lemeshow test with a P >0.05 indicating a good fit.

The model was validated both internally and externally in the training cohort and validation cohort respectively. In the internal validation, bootstrapping with 1000 iterations was used to obtain the Concordance index (C index) of the model, with the greater C index (ranging from 0.5 to 1.0) representing higher predictive accuracy of the model. In the external validation, we evaluated the discriminatory ability of the model in a separate cohort based on ROC-AUC analysis.

Furthermore, we assessed the clinical usefulness of the predictive model by decision curve analysis (DCA). First, we performed DCA to calculate the net benefit of using the nomogram to predict sIA stability on varied probability thresholds. Second, the probability threshold of the optimal cut-off value based on the maximized Youden index was used to estimate the specific predictive value of the model.

## Results

Clinical features of the study population

From January 2017 to December 2021, 613 IAs were diagnosed based on digital subtraction angiography (DSA) at the study hospital. Among 382 sIAs, 352 patients who met the inclusion criteria were enrolled in the study. The enrolled population comprised 215 ruptured sIAs and 167 unruptured sIAs, with a mean age of 58.00 (50-62.25) years for the whole population. The demographic and clinical features, including gender, age, hypertension, blood pressure (BP) at admission, coronary heart disease (CAD), diabetes mellitus (DM), alcohol intake, and smoking, were similar between the ruptured and unruptured groups (p>0.05), but a slightly higher incidence of hyperlipidemia was seen in the ruptured group (p=0.043) (Table 1).

**Table 1:** Demographic and clinical features of patients in ruptured and unruptured groups Unless otherwise indicated, values are expressed as the number of patients (%). BP=blood pressure; SBP=systolic blood pressure; DBP=diastolic blood pressure

Characteristic	Total (n=382)	Ruptured groups (n=215)	Unruptured groups (n=167)	P value
Age in years, M(P_25-_P_75_)	58.00 (50.00-65.25)	57.00 (50.00-65.00)	60.00 (50.00-66.00)	0.507
Sex				0.775
Female	225 (58.9%)	128 (59.5%)	97 (58.1%)	
Male	157 (41.1%)	87 (40.5%)	70 (41.9%)	
BP at admission in mmHg M(P_25-_P_75)_				
SBP	139.00 (124.00-154.00)	140.00 (126.00-158.00)	139.00 (122.00-150.00)	0.081
DBP	81.00 (71.00-89.00)	82.00 (73.00-90.00)	80.00 (70.00-87.00)	0.056
Hypertension	221 (57.9%)	126 (58.6%)	95 (56.9%)	0.736
Hyperlipidemia	75 (19.6%)	50 (23.3%)	25 (15.0%)	0.043
Diabetes mellitus	52 (13.6%)	23 (10.7%)	29 (17.4%)	0.059
Coronary heart disease	10 (2.6%)	3 (1.4%)	7 (4.2%)	0.090
Alcohol intake	55 (14.4%)	28 (13.0%)	27 (16.2%)	0.385
Smoking	105 (27.5%)	56 (26.0%)	49 (29.3%)	0.474

Aneurysm location and morphological characteristics

The location and morphological characteristics of sIA were compared between the ruptured and unruptured groups. There is a significant difference in sIA location, irregularity, daughter dome, multiple lobes, aneurysm size, width, perpendicular height, parent artery, SN, AR, SW, SR, aneurysm volume, and volume/surface area between the two groups (Table 2).

**Table 2:** Morphological features of ruptured and unruptured small intracranial aneurysms ACA: anterior cerebral artery; AcomA: anterior communicating artery; ICA: internal carotid artery; MCA: middle cerebral artery; VA: vertebral artery; BA: basilar artery

Characteristic	Total(n=382)	Ruptured groups(n=215)	Unruptured groups(n=167)	P value
Location of aneurysm				0.041
ACA	19 (5.0%)	11 (5.1%)	8 (4.8%)	
AcomA	84 (22.0%)	72 (33.4%)	12 (7.2%)	
ICA	184 (48.2%)	70 (32.6%)	114 (68.2%)	
MCA	52 (13.6%)	30 (14.0%)	22 (13.2%)	
PcomA	20 (5.2%)	17 (7.9%)	3 (1.8%)	
VA	10 (2.6%)	7 (3.3%)	3 (1.8%)	
BA	13 (3.4%)	8 (3.7%)	5 (3.0%)	
Irregular shapes	170 (44.5%)	119 (55.3%)	51 (30.5%)	<0.001
Daughter dome	66 (17.3%)	55 (25.6%)	11 (6.6%)	<0.001
Multiple lobes	37 (9.7%)	28 (13.0%)	9 (5.4%)	0.012
Aneurysm size, mm	4.59 (3.30-5.68)	4.96 (3.56-5.91)	4.09 (2.89-5.44)	<0.001
Aneurysm width, mm	3.19 (2.40-4.00)	3.24 (2.50-4.19)	3.11 (2.15-3.85)	0.042
Perpendicular height, mm	3.56 (2.64-4.74)	3.86 (2.98-4.96)	3.24 (2.23-4.23)	<0.001
Aneurysm neck, mm	2.58 (2.01-3.36)	2.56 (1.98-3.32)	2.63 (2.02-3.50)	0.416
Parent vessel, mm	2.54 (1.98-3.24)	2.34 (1.90-3.05)	2.74 (2.11-3.58)	<0.001
Size-to-neck ratio	1.62 (1.28-2.04)	1.76 (1.39-2.24)	1.44 (1.17-1.87)	<0.001
Aspect ratio	1.35 (1.01-1.75)	1.43 (1.14-1.91)	1.22 (0.86-1.57)	<0.001
Size-to-width ratio	1.36 (1.16-1.60)	1.39 (1.18-1.73)	1.32 (1.12-1.52)	0.011
Size ratio	1.41 (0.98-1.87)	1.57 (1.15-2.09)	1.19 (0.83-1.58)	<0.001
Aneurysm volume, mm^3^	24.21 (10.77-46.72)	26.43 (12.57-49.81)	21.47 (7.61-41.67)	0.007
Aneurysm surface area, mm^2^	5.23 (3.17-8.88)	5.15 (3.08-8.66)	5.43 (3.20-9.62)	0.416
Volume-to-surface ratio	4.08 (2.24-7.33)	4.75 (2.65-8.68)	3.29 (1.73-6.05)	<0.001

We performed subgroup analysis on aneurysm characteristics in patients with multiple sIA who have at least one ruptured and one unruptured sIA simultaneously. The benefit of this analysis is that the demographic and general medical features could be best matched between the ruptured and unruptured groups. A significant difference was observed between the ruptured groups and unruptured group in irregular shapes, daughter dome, aneurysm width, perpendicular height, parent vessel, SN, SR, AR, SW, aneurysm volume, and volume/surface area, which is consistent with that observed in all studied patients, indicating that those identified aneurysm features were reliable as candidate predictors for rupture of sIA (Table 3).

**Table 3:** Demographic characteristics and morphological features of multiple small intracranial aneurysms ACA: anterior cerebral artery; AcomA: anterior communicating artery; ICA: internal carotid artery; MCA: middle cerebral artery; PcomA: posterior communicating artery; VA: vertebral artery; BA: basilar artery

Characteristics	Total(n=51)	Ruptured groups(n=25)	Unruptured groups(n=26)	P value
Location of aneurysm				0.983
ACA	3 (5.8%）	2 (8.0%)	1 (3.9%)	
AcomA	11 (21.5%)	6 (24.0%)	5 (19.2%)	
ICA	26 (50.9%)	12 (48.0%)	14 (53.8%)	
MCA	7 (13.7%)	3 (12.0%)	4 (15.3%)	
PcomA	0 (0%)	0 (0%)	0 (0%)	
VA	2 (4.0%)	1 (4.0%)	1 (3.9%)	
BA	2 (4.0%)	1 (4.0%)	1 (3.9%)	
Irregular shapes	20 (39.2%)	14 (56.0%)	6 (23.1%)	0.016
Daughter dome	9 (17.6%)	9 (36.0%)	0 (0%)	0.003
Multiple lobes	4 (7.8%)	4 (16.0%)	0 (0%)	0.109
Number of aneurysms				0.076
2	32 (62.7%)	16 (64.0%)	16 (61.6%)	
3	6 (11.8%)	4 (16.0%)	2 (7.7%)	
4	8 (15.7%)	3 (12.0%)	5 (19.2%)	
≥5	5 (9.8%)	2 (8.0%)	3 (11.5%)	
Aneurysm size, mm	3.79±1.57	4.16±1.50	3.44±1.58	0.103
Aneurysm width, mm	2.58 (1.90-3.97)	3.80 (2.46-4.49)	2.14 (1.73-3.23)	0.001
Perpendicular height, mm	2.64 (1.78-4.23)	3.52 (2.15-4.76)	2.09 (1.57-3.42)	0.026
Aneurysm neck, mm	2.53±0.91	2.45±0.84	2.60±0.99	0.571
Parent vessel, mm	2.60±0.99	2.49±0.67	3.00±0.92	0.028
Size-to-neck ratio	1.41 (1.14-1.86)	1.62 (1.32-2.21)	1.21 (1.05-1.53)	0.003
Aspect ratio	1.13 (0.76-1.48)	1.62 (1.32-2.21)	0.89 (0.71-1.27)	0.005
Size-to-width ratio	1.29 (1.10-1.48)	1.34 (1.03-1.96)	1.36 (1.16-1.52)	0.019
Size ratio	1.09 (0.73-1.52)	1.18 (1.03-1.36)	0.81 (0.51-1.28)	0.002
Aneurysm volume, mm^3^	12.25 (5.48-42.54)	1.30 (0.96-1.80)	8.10 (3.55-21.61)	0.003
Aneurysm surface area, mm^2^	4.99 (2.78-8.09)	34.02 (7.85-56.42)	4.80 (2.18-9.32)	0.720
Volume-to-surface ratio	3.02 (1.29-5.94)	4.99 (3.10-7.94)	1.53 (1.03-3.11)	0.000

Selection of risk factors and construction of nomogram

**Figure 2:**
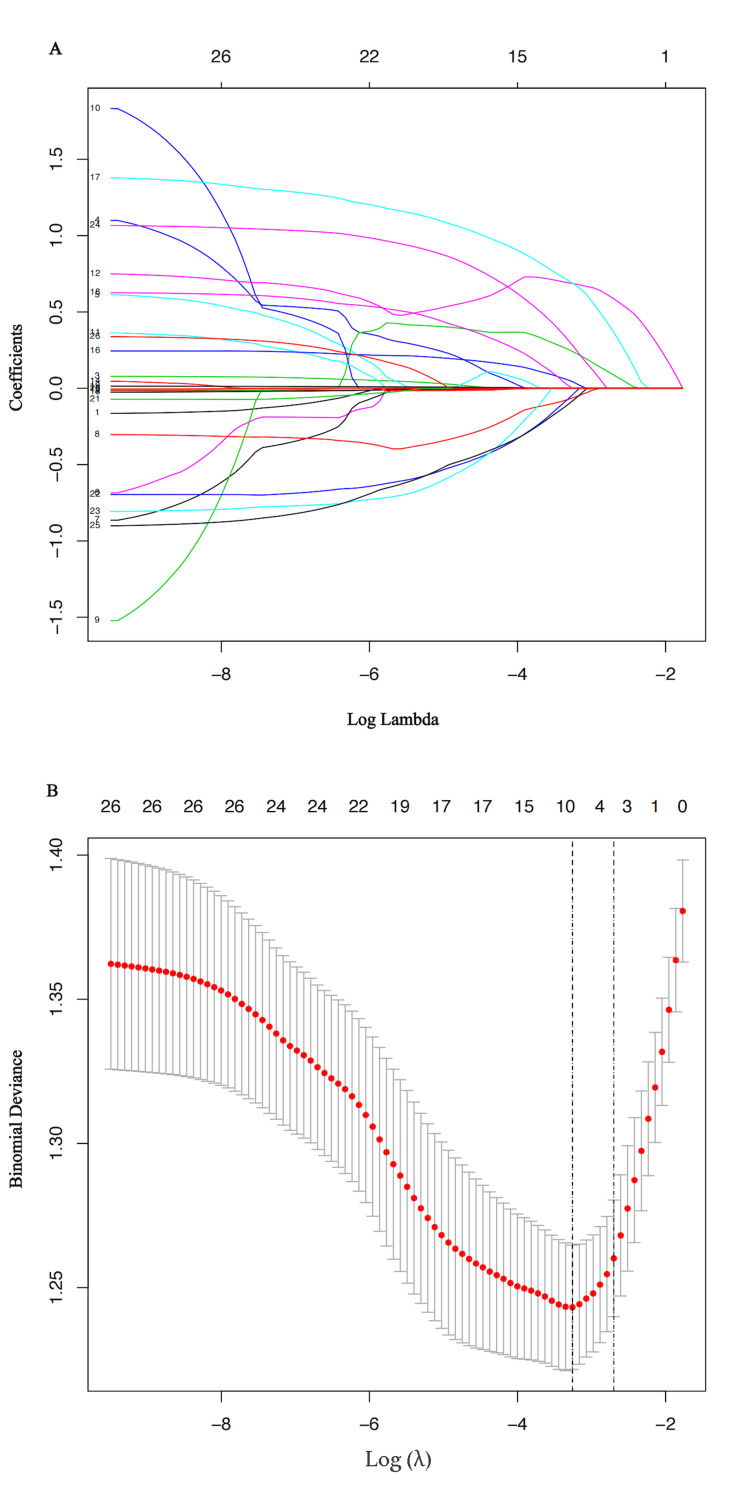
LASSO regression analysis of associated factors (A) The selection of the optimal parameter lambda value using the minimum criterion of 10-fold cross-validation (λ =0.03848165). The LASSO coefficient profile of 26 predictors was drawn based on log (λ). (B) At the minimal value of the mean square error of the classification, the optimal parameter lambda value was drawn, including eight optimal features with nonzero coefficients. The histogram of eight features was presented. LASSO: Least Absolute Shrinkage and Selection Operator

Eight variables from the 26 clinical and morphological factors were selected by LASSO (Least Absolute Shrinkage and Selection Operator) regression analysis in the training cohort, including DM, hyperlipidemia, smoking, SN, SR, diameter of parent artery, irregularity of aneurysm, and daughter dome (Figure 2).

Logistic regression with these eight variables further revealed that hyperlipidemia, daughter dome, SN, and SR were independent risk factors for sIA rupture (Table 4).

**Table 4:** Multivariate logistic regression analysis of the rupture of small intracranial aneurysm

Characteristic	P value	OR	95%CI
Hyperlipidemia	0.008	2.747	1.322-5.956
Size-to-neck ratio	0.021	1.807	1.131-3.063
Size ratio	0.007	2.221	1.262-4.025
Parent vessel	0.163	0.764	0.520-1.111
Irregular shapes	0.327	1.370	0.730-2.576
daughter dome	0.012	3.068	1.311-7.598
Diabetes	0.196	0.575	0.256-1.253
Smoking	0.100	0.586	0.308-1.104

**Figure 3:**
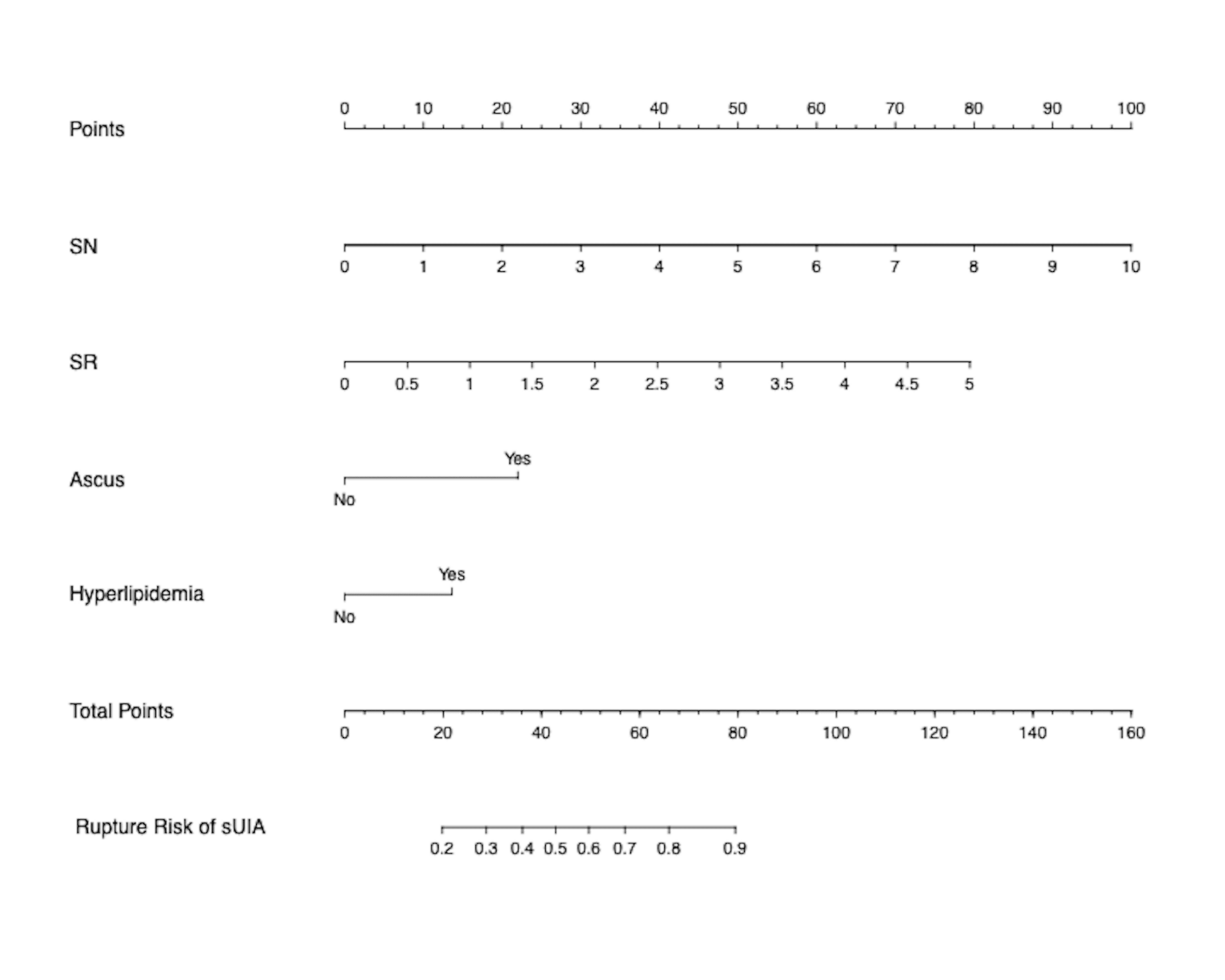
Nomogram of the prediction model in the training cohort Notes: A nomogram of a prediction model was developed in the training cohort, and the SN, SR, and daughter dome. Hyperlipidemia was incorporated. The total score was calculated by adding the score for each risk factor, and then the probability of the rupture of a small intracranial aneurysm was predicted on the risk axis. SN: size-to-neck ratio; SR: size ratio; sUIA: small unruptured intracranial aneurysm

The risk of sIA rupture for patients comorbid with hyperlipidemia was 2.747 (95%CI=1.322~5.956, P=0.008) times higher than that for patients without comorbidity. sIA with a daughter dome has a 3.068 (95%CI =1.311~7.598, P =0.012) times risk for rupture than sIA without a daughter dome. sIA with larger SN (OR=1.807, 95%CI=1.131~3.063, P =0.021) and SR ((OR=2.221, 95% CI=1.262~4.025, P=0.007) showed a significantly higher risk for rupture. A nomogram was constructed based on the four risk factors identified by logistic regression. (Figure 3).

Validation of the nomogram

**Figure 4:**
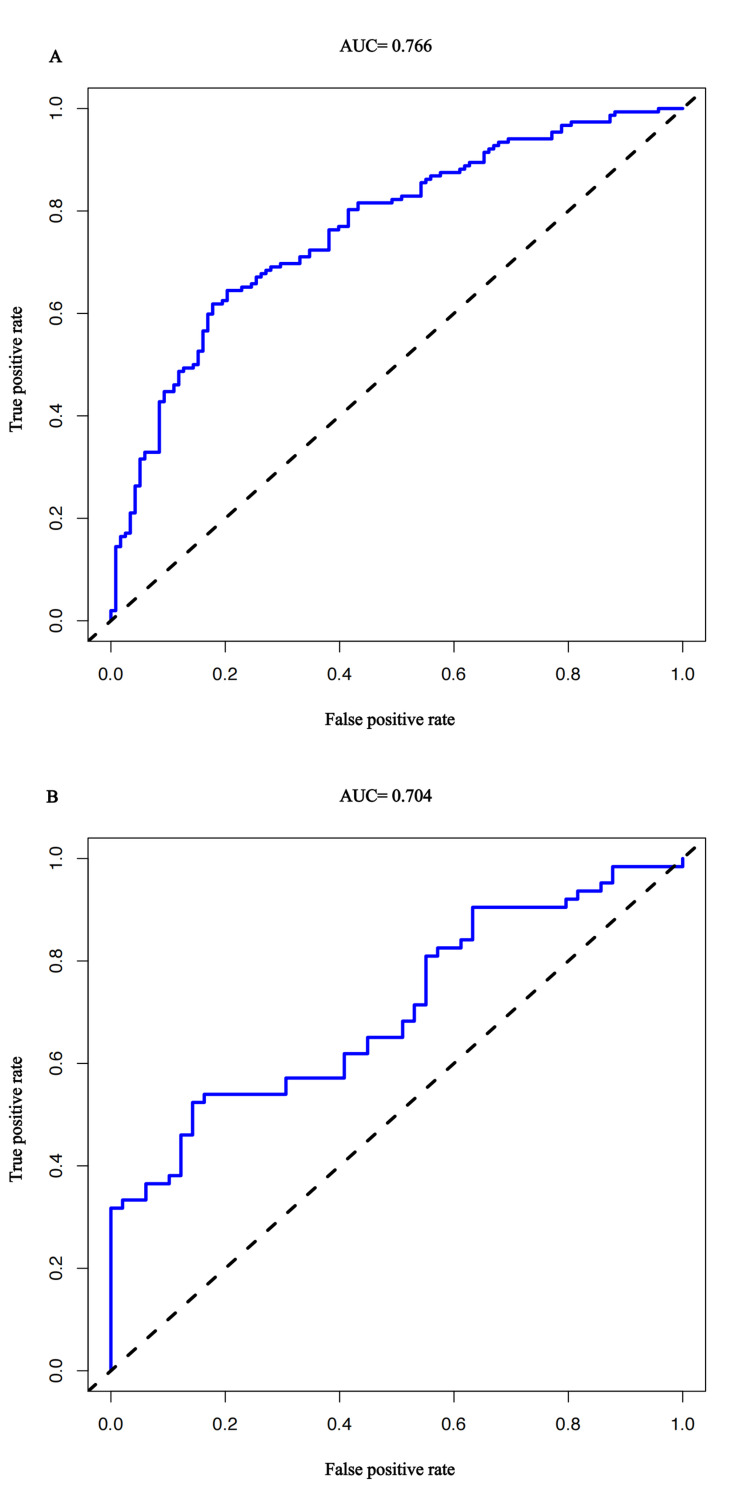
The area under the ROC curve of the training cohort and validation cohort (A) The area under the ROC curve of the training cohort (AUC=0.766). (B) Area under ROC curve of validation cohort (AUC=0.704). ROC: receiver operating characteristic; AUC: area under the curve

To verify the discriminatory ability of the nomogram, the ROC analysis was performed in both the training cohort and the validation cohort separately. The AUC values of the nomograms are 0.766 (95% CI=0.711-0.815) (Figure 4A) and 0.704 (95% CI=0.608-0.810) (Figure 4B) for the training cohort and validation cohort, respectively.

Internal validation by bootstrap sampling showed a C index of 0.756 for the nomogram, indicating good discriminatory ability of the predictive model.

**Figure 5:**
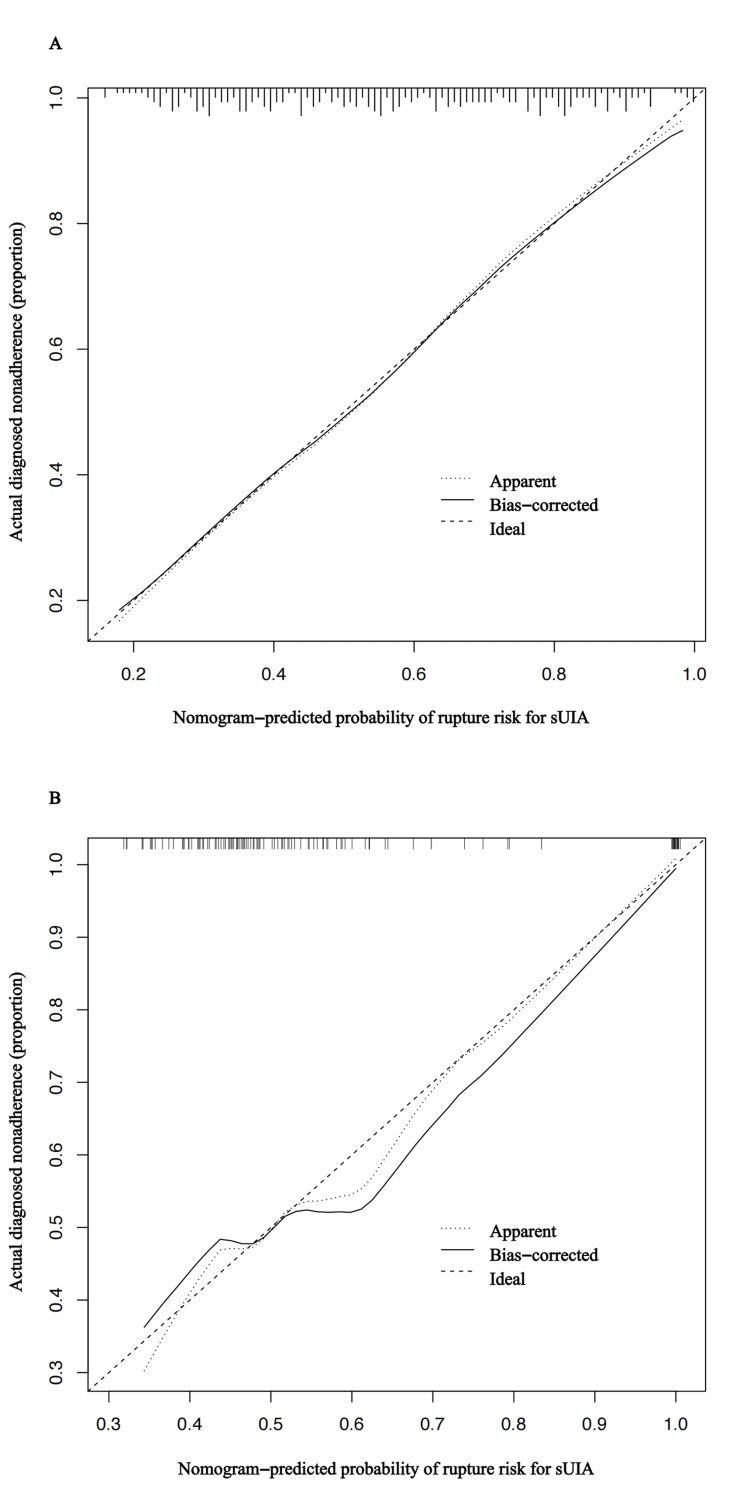
Calibration plot of the prediction model in the training cohort and validation cohort Notes: Calibration curves depict the calibration of the predictive model in terms of the agreement between the predicted risk of sUIA rupture and the observed actual status of sUIA rupture. The abscissa of the calibration curve is the predicted probability and the ordinate is the actual probability. The calibration curves demonstrated good agreement between the nomogram-predicted rupture of sUIA probability and the actual rupture of sUIA probability in the training cohort, indicating the reliability of our nomogram. sUIA: small unruptured intracranial aneurysm

The calibration plot in the training cohort showed good agreement between the predicted risk for sIA rupture and the observed event (Figure 5A) and the Hosmer-Lemeshow test yielded a P value of 0.847, indicating the model was well-fitted. To further verify the efficacy of the development nomogram, we conducted validations in a separate validation cohort. The calibration curve presented in Figure 5B also shows a satisfactory agreement between the estimated and actual risks.

Clinical use of the predictive model

**Figure 6:**
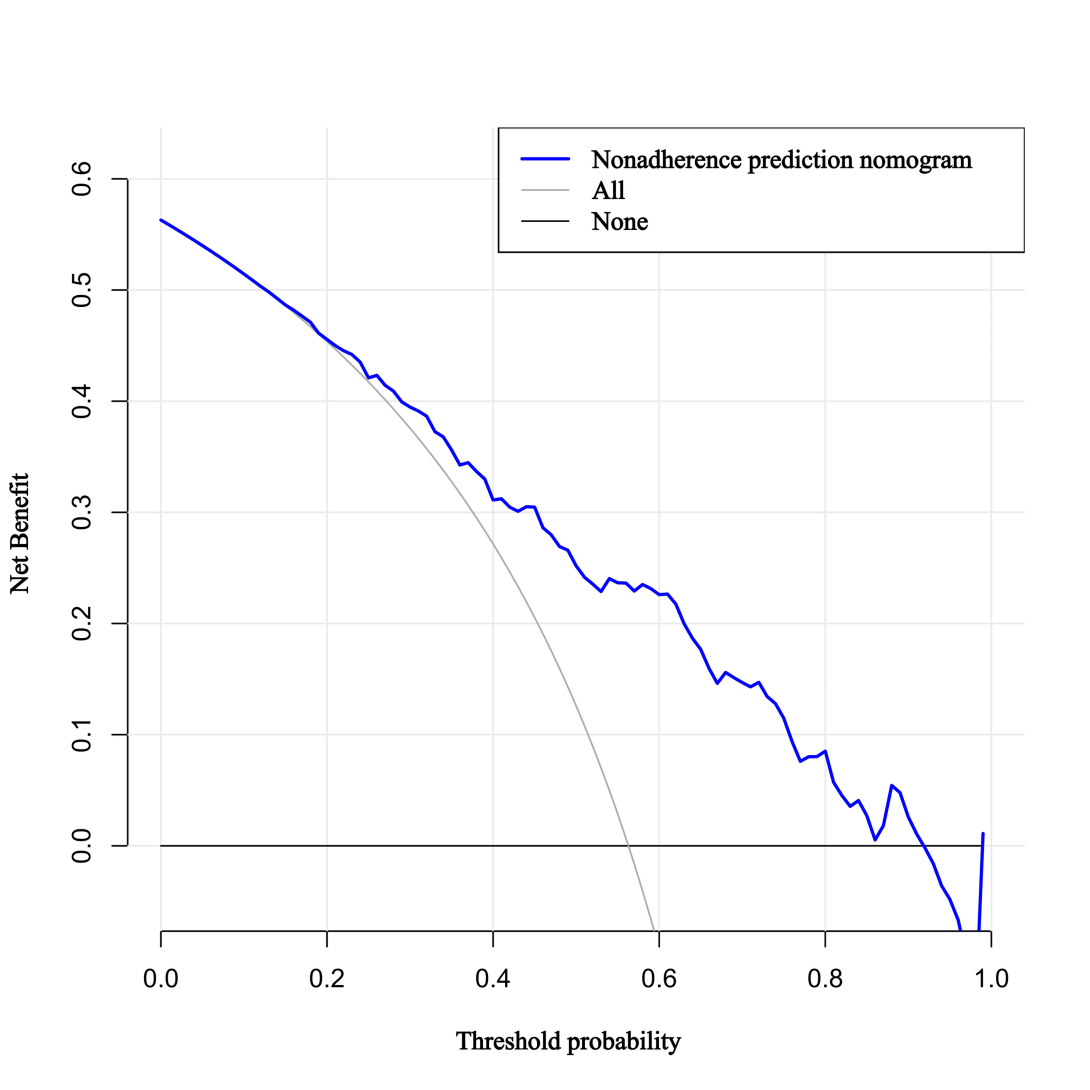
Decision analysis curve of the prediction model in the training cohort and validation The y-axis represents the net benefit while the x-axis stands for the threshold probability. The gray line of “All” refers to the assumption that all sUIA reached the endpoint of rupture, the black line of “none” to the hypothesis that no sUIA reached the endpoint, and the blue line represents the prediction model. The graph gives the expected net benefit per sUIA. The wider range of threshold probabilities of the nomogram indicates a superior net benefit. sUIA: small unruptured intracranial aneurysm

The decision curve analysis was created to assess the clinical usefulness of the predictive model in the training cohort. It was shown that when the threshold probability is in the range of 0.13 to 0.92, a treatment strategy made based on the nomogram will bring more benefit than harm to the patient (Figure 6).

We further explore the magnitude of benefit on the optimal threshold of probability determined to be 0.582 in the ROC analysis. It is demonstrated that when this threshold probability is applied, the predictive model will achieve a net benefit of 0.25, which means that 25 out of 100 sIA patients will benefit by using the nomogram, indicating the sound clinical usefulness of the predictive model.

## Discussion

In the current study, we developed a predictive model for sIA rupture based on four patient and aneurysmal characteristics of easy accessibility. Validation of the nomogram by discrimination, calibration, and decision curve analysis (DCA) showed good reliability and clinical utility of the predictive model for risk stratification of sIA stability, indicating a satisfactory potential of this nomogram-based model for guiding clinical management of unruptured sIA.

The incidence of intracranial aneurysms is estimated to be as high as 3.2% in the general population [14]. Surgical repair is the most effective way to prevent rupture of an intracranial aneurysm, but it is still inconclusive whether unruptured aneurysms smaller than 7 mm in diameter should be repaired prophylactically by surgical clipping or endovascular coiling since the majority of sIA may remain stable for long [2-3]. Previous studies have demonstrated that the risk of aneurysm rupture can be stratified according to features such as age, aneurysm size, history of SAH, and BP, but most of these investigations were performed in patients with aneurysm of varied sizes, thus the results may not be well applied to sIA [15-16]. Data on the efficacy of sIA-specific risk predictive models is still limited. By screening the clinical features of sIA, we found that hyperlipidemia was the independent risk factor for sIA rupture, which was inconsistent with most previous studies that showed no association between hyperlipidemia and aneurysm rupture [9-11]. It is noted that a recent study showed that dyslipidemia was a protective factor for the rupture of an aneurysm smaller than 7 mm in the anterior circulation [17]. Some have argued that the vessel stiffness attributed to atherosclerosis in dyslipidemia may protest against aneurysm rupture. But atherosclerotic plaques also lead to the weakening of the vessel wall by local oxidative or inflammatory injuries and contribute to the formation and rupture of aneurysm in the affected artery. The other explanation for the inconsistency between our study and previous ones may be due to the protective effect of statins on the vascular endothelium, which increases the resistance of the aneurysm to local wall shear stress. Further investigation with stratification of the degree of atherosclerotic damage of the parent vessel as well as the use of statin by the patient are needed to elucidate the association between dyslipidemia and risk of sIA rupture. Older age, female gender, and uncontrolled hypertension were reported to be associated with an increased risk of aneurysm rupture [2,17], which was inconsistent with that observed in our study. We found the absence of association of these features with an sIA rupture. A similar result was reported in a recent study of IA smaller than 7 mm. This indicated that the effect of these clinical features on aneurysm rupture may be influenced by IA size and the small sample size. Further investigation with a larger sample size and prospective design was needed to make clear the specific clinical features that are independently associated with the rupture of sIAs.

With respect to the IA features, irregular shape, larger SN, and larger SR were selected by regression analysis as risk factors for the predictive nomogram. The irregular shape of an aneurysm, usually defined as the presence of a daughter dome, multiple lobes, or aneurysm wall protrusions, was consistently observed to be associated with an increased risk of IA rupture, regardless of IA size and location [16]. Koopman et al. reported that irregular shape was an independent risk factor for the rebleeding of a ruptured IA after adjustment for other patient and aneurysmal features, including age, sex, Prognosis on Admission of Aneurysmal Subarachnoid Hemorrhage (PAASH) score, aneurysm location or size and aspect, and bottleneck ratio [18]. Recently, using four-dimensional CT angiography, Zhou et al. reported that larger SR and irregular shape were among the indicators of irregular pulsation of sIA smaller than 7 mm in diameter, which was independently associated with rupture of small IA [19]. It is estimated that due to its irregular shape, the IA was exposed to a more unstable hemodynamic environment, in terms of faster blood flow, higher wall shear stress, more oscillatory shear, varied inflow jet, and multiple vortices, which contributed to the high risk of rupture or growth of IA [16].

The association of SR and SN with small IA rupture was not as consistent in previous studies as that for the irregular shape of IA. Zhou et al. found that larger SR was associated with the rupture of small IA less than 7 mm in diameter [19]. Other studies have reported similar results [7,20]. However, a recent study showed that SR was not an independent risk factor [9]. Inconclusive results were also observed for the association between SN and IA rupture. Nevertheless, several mechanisms may underlie the effect of larger SR or SN and are supportive of their association. 

Larger SR means that the diameter of the parent vessel is small, indicating a relatively thin vessel wall that is more vulnerable to local hemodynamic challenges. A study further showed that compared with an IA with SR smaller than 2, a complex blood-flow vortex was more common in the sac of an IA with SR larger than 2, which was significantly associated with an increased risk of IA rupture [21]. Another investigation also showed that a larger SR predicted the rupture of an IA smaller than 5 mm in diameter [22]. It should be noted that increased SR may be contributed simultaneously by vasospasm of the parent artery following SAH, thus a repeated angiography to evaluate the diameter of the parent vessel should be performed in further investigations of SR of the rupture IA. 

The length-to-width of neck ratio (SN) is another predictor for small IA rupture identified in our study, which is consistent with that reported by Lauric et al. [23]. Previous data on the association between SN and the risk of aneurysm rupture is relatively limited [23]. More consistent results have been seen in the influence of AR, which represents similar morphological features with SN, and in some research, the identical definition with SN was used for AR [13,24]. It was hypothesized that larger SN and AR contributed to a higher risk of IA rupture through shared mechanisms. It was demonstrated that the height of the IA dome and the width of the neck determined the intra-aneurysm flow volume and velocity, which may be related to injurious stress on the aneurysmal wall, such as local inflammation or hypoxia, which led to the rupture of the IA [13,25].

Following the extensive use of non-invasive brain angiographic techniques, a growing number of unruptured small IA were identified in asymptomatic individuals. Uncertainty continues to exist for the optimal management of unruptured small IAs due to the lack of a promising predictive model for IA stability. Evidence showed that early proposed predictive scoring methods that developed from IA of non-specific sizes may not be well-applied for small IA. A recent study reported that the majority of ruptured IA were small in size and with low rupture risk scores on calculators developed by early investigations, including the International Study of Unruptured Intracranial Aneurysms (ISUIA), Unruptured Cerebral Aneurysm Study (UCAS), and PHASES (population, hypertension, age, size of the aneurysm, earlier subarachnoid hemorrhage (SAH) from another aneurysm, and site of the aneurysm) [26]. In this context, some recent efforts have been made to develop a predictive model for small IA rupture [9,10,27,28]. A nomogram was constructed in most of these studies since it offered a clear, simplified scoring system incorporating both clinical and aneurysmal features, which would be easy to understand and use in clinical settings. Zhu et al. reported a nomogram-based model constructed with five clinical and morphological risk factors for small IA less than 7 mm in diameter, the model yielded a sensitivity and specificity of 82.6% and 69.3%, respectively, with an AUC value of 0.803 in the developing cohort [9]. In the study of Lou et al., the nomogram developed with six clinical and aneurysmal features showed superior sensitivity and specificity over the PHASES scoring system in small IA less than 5 mm [10]. Similarly, with the four selected, easy-to-access factors, the current model also achieved sound sensitivity and specificity of 64.47% and 79.66%, respectively, at the calculated optimal cut-off value, indicating a good predictive value. Noteworthy, some inconsistent findings from previous reports were seen in our study. First, most previous studies showed that the risk of rupture increased with aneurysm size, but the current study found that the size of sIA was not significantly different between the ruptured and unruptured groups. We infer that the influence of IA size may vary by the size of the IA, which is supported by the results reported by Zhu et al. in sIA [9]. Second, the significant association between IA location and risk of rupture was conclusively reported by previous studies, but this factor was not selected as a risk predictor in our study. One possible explanation may be there were few cases with IA located in posterior circulation in the current investigation. Thus, further study enrolling more posterior IAs should be performed to clarify whether IA location is a promising predictor for constructing the predictive model.

Limitations

Some limitations were seen in our study. First, the study is of retrospective design and the outcomes may be influenced by many factors, such as comorbidity, medication, mode of routine care, and so on, thus a prospective design should be used in future investigations. Second, the sample size is relatively small in our study; based on the current results, the future prospective study should enroll more patients, especially with IA in the posterior circulation to increase the reliability of observations.

## Conclusions

Taken together, we developed and validated a risk-predictive model for sIAs with a diameter of less than 7 mm. The model demonstrates strong predictive capacity and holds significant potential for clinical application. Specifically, this model can aid clinicians in risk stratification of patients with small intracranial aneurysms, thereby assisting in the decision-making process regarding the need for closer monitoring or early intervention. Future prospective studies are warranted to further explore and validate the model's effectiveness in diverse clinical settings and to compare its performance with other recently developed predictive models.

## Figures and Tables

**Figure 7:**
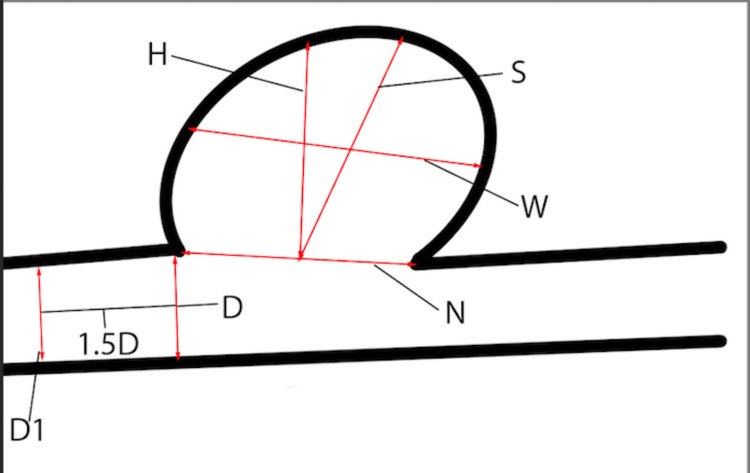
Measurement of aneurysm morphological features S, maximum diameter of aneurysm; H, perpendicular height; W, aneurysm width; N, aneurysm neck diameter; D: diameter of the parent artery at the edge of the neck; D1: the diameter of the parent vessel perpendicular to flow, measured at 1.5D from D This figure is the original work of the authors.

## Appendices

Appendix A

Appendix B

S1 Method: Definitions of Morphological Features and Formulas

Aneurysm size: defined as the maximum distance from the center of the aneurysm neck to the dome of the aneurysm.

Perpendicular height: defined as the maximum perpendicular distance of the dome from the aneurysm neck plane.

Aneurysm width: the maximum distance of the dome perpendicular to the maximum height.

Aneurysm neck diameter: defined as the maximum diameter of the aneurysm neck plane.

Diameter of the parent artery: defined as the mean value of D and D1.

Aneurysm volume: the volume of aneurysm. Calculate according to the following formula: V=W2/6*D*π2 

Surface area: the surface of the aneurysm neck plane. Calculate according to the following formula: S=π*（N/2）2

Aspect ratio (AR): defined as the ratio of the perpendicular height to neck diameter.

Size ratio (SR): defined as the aneurysm/diameter of the parent artery.

Size-to-neck ratio: defined as the ratio of aneurysm size to neck ratio.

Size-to-width ratio: defined as the ratio of aneurysm size to width ratio.

Volume-to-surface ratio: defined as the ratio of aneurysm volume to surface area ratio.
